# A case of vasculitis case with unusual renal pathological findings presenting with rapidly progressing renal failure

**DOI:** 10.1002/ccr3.3277

**Published:** 2020-09-07

**Authors:** Hyo Jung An, Ha Nee Jang, Tae Won Lee, Changhyo Yoon, Dong Jun Park, Eunjin Bae

**Affiliations:** ^1^ Department of Pathology Gyeongsang National University Changwon Hospital Changwon Korea; ^2^ Department of Internal Medicine Gyeongsang National College of Medicine and Gyeongsang National University Hospital Jinju Korea; ^3^ Department of Internal Medicine Gyeongsang National University College of Medicine and Gyeongsang National University Changwon Hospital Changwon Korea; ^4^ Department of Neurology Gyeongsang National University Changwon Hospital Gyeongsang National University School of Medicine Jinju Korea

**Keywords:** acute kidney injury, antineutrophil cytoplasmic antibody‐associated vasculitis, plasma cell

## Abstract

Plasma cell infiltration may be an early lesion during renal invasion by ANCA‐associated vasculitis (AAV), and AAV should be considered in the differential diagnosis with acute renal failure and systemic symptoms.

## Graphical abstract

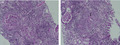

## 1 INTRODUCTION

Antineutrophil cytoplasmic antibody (ANCA)‐associated vasculitis (AAV) is defined as necrotizing vasculitis, with few or no immune deposits, predominantly affecting the small vessels, and associated with myeloperoxidase (MPO) ANCA or proteinase 3 (PR3) ANCA. Not all patients have ANCA.^1^ AAV is categorized into four variants based on clinical manifestations and histological findings, microscopic polyangiitis (MPA), granulomatosis with polyangiitis (GPA), eosinophilic granulomatosis with polyangiitis EGPA), and single‐organ AAV. MPA has no granulomatous inflammation compared to GPA and EGPA and mainly affects small vessels. EGPA, unlike GPA, has eosinophil‐rich necrotizing granulomatous inflammation and is accompanied by asthma and eosinophilia. Among these entities, glomerulonephritis occurs very often in patient with MPA, commonly in those with GPA and occasionally in those with EGPA.^1^, ^2^ The most typical histological finding of renal AAV is necrotizing glomerulonephritis with crescents affecting few to many glomeruli.^3^ A previous study reported few cases of renal AAV that present with severe tubulointerstitial nephritis (TIN) by plasma cells without underlying plasma‐cell proliferative disease or IgG4‐associated disease. However, this study focused primarily on the pathological findings, and there was only one case in which plasma cells occupied most of the tubulointerstitium, and no detailed clinical features were described. Here, we report a case of renal AAV with rapidly progressing renal failure and unusual histopathological findings in a kidney biopsy.

## 2 CASE REPORT

**FIGURE 1:**
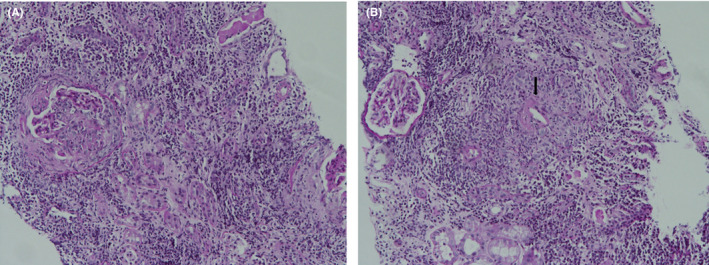
Light microscopic images using periodic acid Schiff (PAS) staining on renal biopsy. Numerous plasma cells and several neutrophils infiltrating to the interstitial area (A) (×100), and small vessels with neutrophil infiltration and fibrin deposition (arrow), indicating necrotizing vasculitis (B) (×100)

**FIGURE 2:**
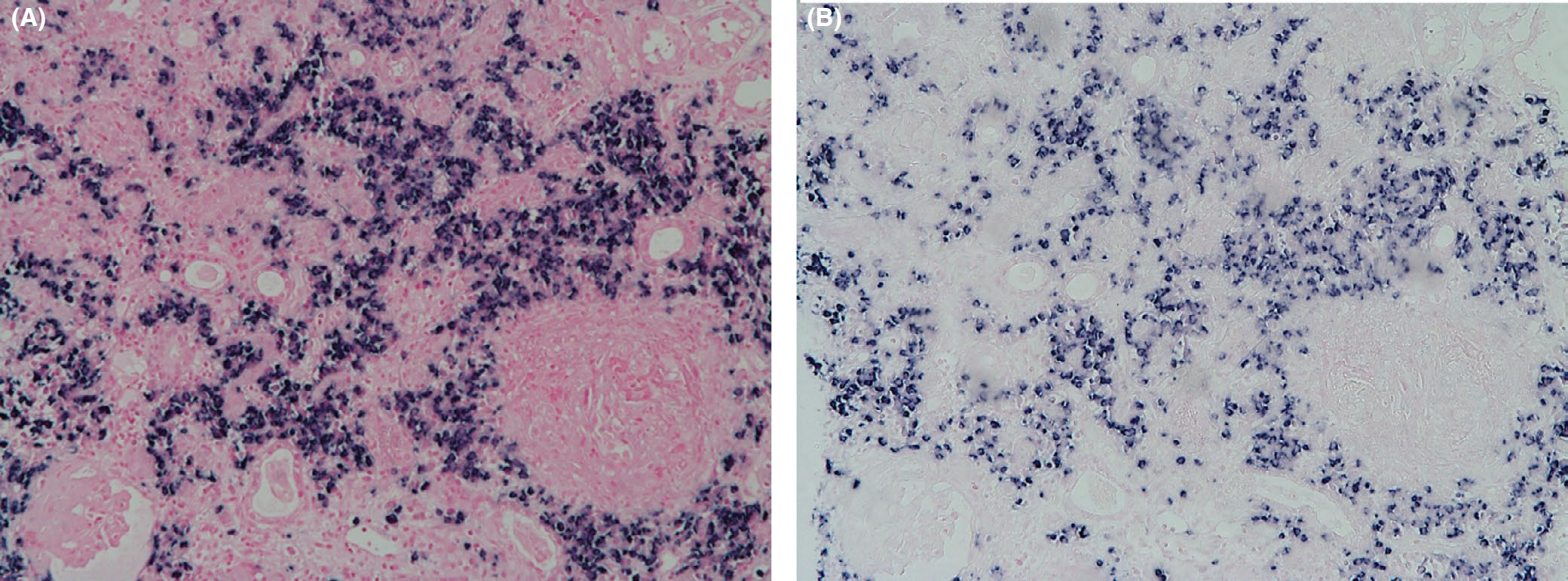
Bone marrow biopsy finding. Both kappa (A) (×100) and lambda (B) show positive in situ hybridization staining pattern, indicating polyclonality. (kappa and lambda in situ hybridization)

A 69‐year‐old man was admitted to the hospital because of anorexia and fever. He had suffered an acute middle cerebral artery territory stroke 45 days previously and underwent treatment. The only medications he was taking were for stroke (aspirin 100 mg, cilostazol 100 mg, atorvastatin 80 mg, and rabamipide 100 mg). On the initial physical examination, his vital signs were as follows: blood pressure, 162/79 mm Hg; pulse rate, 104 beats/min; respiratory rate, 20 breaths/min; and temperature, 37.8°C. He had severe dysarthria and right extremity weakness due to the stroke. No malar rash, oral ulcers, jugular venous distension, or lymphadenopathy were observed. Bilateral fine crackles were detected on chest auscultation, and his abdomen was soft but there was no organomegaly. Multiple pinkish reticulated patches were observed on the lower leg. The results of initial laboratory tests were as follows: white blood cell (WBC) count, 17 630/μL (4000‐10 000/μL); hemoglobin, 9.1 g/dL (13.0‐17.0 g/dL); blood urea nitrogen, 24.3 mg/dL (6‐20 mg/dL); creatinine, 1.52 mg/dL (0.6‐1.2 mg/dL); C‐reactive protein, 153.5 mg/dL (0.0‐5.0 mg/dL); MPO–ANCA, 100.0 U/mL (0‐4.9 U/mL); and PR3–ANCA, negative. The urinalysis revealed the following findings: protein, 2+; blood, 2+; red blood cells, 5‐9/high power fields (HPF); and WBC, 5‐9/HPF. Serum/urine protein electrophoresis and immunofixation showed nonspecific findings (Table 1). Pulmonary interstitial fibrosis was noted on a chest X‐ray (CXR). Brain magnetic resonance imaging revealed no evidence of a newly developed infarction, but the hemorrhagic transformation of the left MCA infarction lesion was detected (frontoparietal lobes, basal ganglia, and insular region). His baseline serum creatinine was 0.71 mg/dL, which increased to 1.52 and 2.24 mg/dL on days 1 and 3 of admission, respectively. A percutaneous renal biopsy was performed on day 7 of admission. Seven fibrocellular crescents out of 17 glomeruli were observed on light microscopy (Figure 1A). Some were associated with fibrinoid necrosis, and numerous neutrophils were detected in the capillary lumen. In addition, a few small vessels with neutrophil infiltration and fibrin deposition were evident, indicating acute necrotizing vasculitis (Figure 1B). Most of the interstitial area was infiltrated by numerous plasma cells and several neutrophils. The possibility of crescentic glomerulonephritis, associated with plasma‐cell proliferative disease or IgG‐related disease, was considered. Kappa and Lambda in situ hybridization revealed a positive staining pattern, indicating polyclonality (Figure 2A,B). In addition, IgG4‐positive plasma cells were up to 200/HPF in the interstitial area. Unlike IgG4‐tubulointerstitial nephritis, there was no sclerosing lymphoplasmacytic inflammation or apparent obliteration of the tubules. Therefore, the patient was diagnosed with microscopic polyangiitis having pauci‐immune crescentic glomerulonephritis with fibrinoid necrotizing vasculitis. A bone marrow (BM) aspiration and biopsy showed a normocellular marrow with no increase in plasma cells (1.8% of all nucleated cells), and no evidence of BM involvement by monoclonal plasma cells. His Birmingham vasculitis activity score was 27. Serum creatinine peaked at 3.1 mg/dL. He received three pulses of methylprednisolone (500 mg/day) followed by prednisone (1 mg/kg/day) and cyclophosphamide (1.5 mg/kg/day). After treatment, his serum creatinine improved to 2.3 mg/dL, and the microscopic hematuria disappeared. Furthermore, his fever and skin lesions improved. He was discharged with prednisolone 40 mg/day (1 mg/kg/day), peroral cyclophosphamide 50 mg/day, and his renal function remained stable until 2 months after discharge. No side effects were observed due to the immunosuppressive agents. The dose of azathioprine was maintained, and the dose of prednisolone was tapered to 30 mg/day. He was subsequently lost to follow‐up and died 16 months later due to pneumonia.

**TABLE 1:** Laboratory findings

Variables	Prior to admission	Post‐admission day 1	Post‐admission day 14	Follow‐up Post discharge 2 mo
WBC (/μL)	10 540	17 630	11 460	14 590
Hemoglobin (g/dL)	13.3	9.1	10.8	11.8
Platelet (/μL)	356 000	531 000	443 000	384 000
Blood urea nitrogen (mg/dL)	8.9	24.3	57.8	50.7
Cholesterol (mg/dL)	94	71	114	140
Total Protein (g/dL)	7.5	6.6	6.5	6.9
Albumin (g/dL)	3.6	2.0	3.1	3.6
Uric acid (mg/dL)	7.7	4.5	8.1	8.2
Creatinine (mg/dL)	0.81	1.52	3.11	2.27
eGFR (mL/min/1.73 m^2^)	100.1	48.4	21.2	30.5
CRP (mg/dL)	14.2	136.8	48	8.7
Antinuclear antibody	‐	Weak positive (1:40)	‐	‐
MPO‐ANCA	‐	Positive (100.0)	‐	‐
PR3‐ANCA	‐	Negative (1.5)	‐	‐
Anti ds‐DNA antibody IgG	‐	Negative (8.6)	‐	‐
Anti ds‐DNA antibody IgM	‐	Negative (12.7)	‐	‐
Serum immunofixation electrophoresis	‐	Negative	‐	‐
Serum, urine protein electrophoresis	‐	Negative		
Urine protein	Trace	4+	2+	‐
Urine RBC (/HPF)	>100	10‐19	10‐19	<1

Abbreviations: CRP, C‐Reactive Protein; eGFR, estimated glomerular filtration rate; HPF, high‐power filed; MPO‐ANCA, myeloperoxidase‐Antineutrophil cytoplasmic antibodies; PR3, proteinase‐3; WBC, White blood cells.

## 3 DISCUSSION

We report a case of unusual kidney biopsy findings with systemic involvement of AAV in an older patient who had recently been treated for cerebral infarction. AAV with severe interstitial plasma cell infiltration without underlying plasma‐cell proliferative disease or IgG4‐associated disease is very rare. In addition, unlike previous cases, this case presented with the clinical features of active vasculitis, such as serological assessment (ANCA testing), fever, cutaneous lesions, and pulmonary fibrosis. Masuzawa et al described 20 cases of ANCA glomerulonephritis (GN) focusing on plasma cell infiltration. They suggested that plasma cell infiltration in the tubulointerstitium might be associated with an early period of renal inflammation. However, they did not show an association between the plasma cell ratio and the kidney prognosis.^4^ Several cases with severe tubulointerstitial injury, as compared to glomerular lesions in ANCA‐GN, have been reported, most of which improved renal function.^5^, ^6^, ^7^ Interestingly, some studies have reported transformation from tubulointerstitial nephritis to crescentic glomerulonephritis in patients with ANCA‐GN. These findings suggest that tubulointerstitial injury in ANCA‐GN, particularly plasma cell infiltration, might be the early, acute lesion. Also, kidney prognosis improves if the disease is found early and treated adequately and quickly. The pathogenesis of AAV is currently not fully understood, but ANCA IgG is considered a major pathogenic factor.^3^, ^8^, ^9^ ANCAs are autoantibodies with epitope specificity against antigens present in the cytoplasm of neutrophils and monocytes. The formation of MPO and PR3‐ANCA might depend on various predisposing factors such as a microbial infection (*Staphylococcus aureus* or *Escherichia coli* infection), genetic factors (PR3‐ANCA with human leukocyte antigen [HLA]‐DP, or MPO‐ANCA with HLA‐DQ), environmental factors (silica), and therapeutic drugs (propylthiouracil).^10^ Primed neutrophils activated by MPO, and PR3‐ANCA lead to degranulation, which releases the contents of the cytoplasmic granules into the surrounding tissues. This process also results in the production of reactive oxygen radicals (RORs), and the release of the granular proteins and the RORs cause tissue injury.^11^ The degranulated neutrophils ultimately die by apoptosis and necrosis. In addition, activation and degranulation of a large number of neutrophils lead to their accumulation and damage to the vessel wall. Leakage of serum proteins and the formation of fibrin give rise to fibrinoid necrosis.^10^ The stimulation of neutrophils by ANCA causes the release of factors that activate complement via an alternative pathway, which could aggravate AAV.^12^ A previous study on B‐cell immunity and AAV reported that the B‐cell autoimmune response be facilitated by impaired T‐ and B‐cell regulation and by B‐cell–stimulating factors released by activated neutrophils.^13^ Interestingly, our case, diagnosed with AAV and severe interstitial plasma cell infiltration and fibrinoid necrosis of the vessels, supported a previous study^14^ regarding the contributions of B cells and plasma cells to ANCA vasculitis. Rituximab has been approved as an AAV therapy, and previous studies have reported that bortezomib inhibits anti‐MPO–mediated necrotizing MPO‐specific plasma cells in the spleen and BM.^14^, ^15^ The possibility of applying AAV therapies (either rituximab or bortezomib) has been suggested, as there are many plasma cells to target. Our case infers an association between B‐cell immunity and AAV, but we could not prove this speculation. Therefore, further research on the involvement of B‐cell immunity and the process of renal injury resulting from ANCA is needed. Ischemic cerebral infarction caused by AAV is rare and is typically resistant to antiplatelet therapy but tends to recur without proper immunosuppressive therapy.^16^ Although stroke in our case was due to an intracranial artery occlusion separate from AAV, AAV can cause neurological manifestations including stroke. Thus, clinicians should consider AAV as cause of acute kidney injury with neurological symptoms. The strength of our study was the unusual histopathological findings of renal involvement of AAV. Based on these results, we deduced various mechanisms of damage to kidney tissue by ANCA. However, the limitation of our study is that we reported only one case, and there was no histopathological confirmation of involvement by other organs in either case. Although we could not obtain the histopathological diagnosis of other organs, we diagnosed this case as AAV by clinical, serological assessment (ANCA testing), and histopathological findings of vascular injury on a renal biopsy.

## 4 CONCLUSION

We report a case of vasculitis that showed acute systemic symptoms with pauci‐immune crescentic glomerulonephritis and plasma cell‐rich infiltrates. Further research is needed on the relevance of B‐cell immunity and the organ damage caused by ANCA.

## CONFLICT OF INTEREST

The authors declare no conflicts of interest.

## AUTHOR CONTRIBUTIONS

HJA: collected data and drafted the manuscript. HNJ: collected data, TWL: interpretation of data, CY and DJP: revised the manuscript. EB: involved in conceptualization, followed up the patient, and drafted the manuscript.

### ETHICAL APPROVAL

Approval was obtained from our Institutional Review Board (No. GNUCH 2019‐08‐029) for this case report, with a waiver of informed consent.

## References

[1] Jennette JC , Falk RJ , Bacon PA , et al. 2012 revised international Chapel Hill consensus conference nomenclature of vasculitides. Arthritis Rheum. 2013;65(1):1‐11. 23045170 10.1002/art.37715

[2] Jennette JC , Nachman PH . ANCA glomerulonephritis and vasculitis. Clin J Am Soc Nephrol. 2017;12(10):1680. 28842398 10.2215/CJN.02500317 PMC5628710

[3] Appel GB , Radhakrishnan J , D'Agati V . Secondary glomerular disease Brenner and Rector's the kidney. Small vessel vasculitis 1 10th ed. Philadelphia: Elsevier 2015;1109‐1118.

[4] Masuzawa N , Nishimura A , Mihara Y , Tamagaki K , Konishi E . Clinicopathological analysis of ANCA‐associated glomerulonephritis focusing on plasma cell infiltrate. Clin Exp Nephrol. 2019;23(12):1373‐1381. 31485791 10.1007/s10157-019-01785-8

[5] Wen YK , Chen ML . Transformation from tubulointerstitial nephritis to crescentic glomerulonephritis: an unusual presentation of ANCA‐associated renal vasculitis. Ren Fail. 2006;28(2):189‐191. 16538980 10.1080/08860220500531559

[6] Lin Z‐S , Liu X‐L , Cui Z , et al. Acute tubulointerstitial nephritis with germinal centers in antineutrophil cytoplasmic antibody‐associated vasculitis: a case report and literature review. Medicine. 2019;98(48):e18178. 31770269 10.1097/MD.0000000000018178 PMC6890356

[7] Plafkin C , Zhong W , Singh T . ANCA vasculitis presenting with acute interstitial nephritis without glomerular involvement. Clin Nephrol Case Stud. 2019;7:46‐50. 31346511 10.5414/CNCS109805 PMC6657422

[8] Jennette JC , Falk RJ . The role of pathology in the diagnosis of systemic vasculitis. Clin Exp Rheumatol. 2007;25(1 Suppl 44):S52‐S56. 17428368

[9] Jennette JC . Nomenclature and classification of vasculitis: lessons learned from granulomatosis with polyangiitis (Wegener's granulomatosis). Clin Exp Immunol. 2011;164(Suppl 1):7‐10. 21447122 10.1111/j.1365-2249.2011.04357.x PMC3095856

[10] Al‐Hussain T , Hussein MH , Conca W , Al Mana H , Akhtar M . Pathophysiology of ANCA‐associated vasculitis. Adv Anat Pathol. 2017;24(4):226‐234. 28537941 10.1097/PAP.0000000000000154

[11] Söderberg D , Segelmark M . Neutrophil extracellular traps in ANCA‐associated vasculitis. Front Immunol. 2016;7:256. 27446086 10.3389/fimmu.2016.00256 PMC4928371

[12] Xiao H , Schreiber A , Heeringa P , Falk RJ , Jennette JC . Alternative complement pathway in the pathogenesis of disease mediated by anti‐neutrophil cytoplasmic autoantibodies. Am J Pathol. 2007;170(1):52‐64. 17200182 10.2353/ajpath.2007.060573 PMC1762697

[13] Jennette JC , Falk RJ . B cell‐mediated pathogenesis of ANCA‐mediated vasculitis. Semin Immunopathol. 2014;36(3):327‐338. 24777746 10.1007/s00281-014-0431-y PMC4084547

[14] Bontscho J , Schreiber A , Manz RA , Schneider W , Luft FC , Kettritz R . Myeloperoxidase‐specific plasma cell depletion by bortezomib protects from anti‐neutrophil cytoplasmic autoantibodies‐induced glomerulonephritis. J Am Soc Nephrol. 2011;22(2):336‐348. 21233415 10.1681/ASN.2010010034 PMC3029906

[15] Schrezenmeier E , Jayne D , Dörner T . Targeting B cells and plasma cells in glomerular diseases: translational perspectives. J Am Soc Nephrol. 2018;29(3):741‐758. 29326157 10.1681/ASN.2017040367 PMC5827591

[16] Zheng Y , Zhang Y , Cai M , Lai N , Chen Z , Ding M . Central nervous system involvement in ANCA‐associated vasculitis: what neurologists need to know. Front Neurol. 2019;9:1166. 30687221 10.3389/fneur.2018.01166 PMC6335277

